# Supplementary material for: The SARS-CoV-2 Spike Protein Mutation Explorer: Using an Interactive Application to Improve the Public Understanding of SARS-CoV-2 Variants of Concern
Source: J Vis Commun Med. Author manuscript; Available in PMC 2025 Feb 24. (PMC10726978; doi:10.1080/17453054.2023.2237087)
Supplement: Supplementary Material [file EMS192801-supplement-Supplementary_Material.docx]

The SARS-CoV-2 Spike Protein Mutation Explorer: using an interactive application to improve the public understanding of SARS-CoV-2 variants of concern

Sarah Iannucci, William T. Harvey, Joseph Hughes, David L. Robertson, Matthieu Poyade, and Edward Hutchinson

JOURNAL OF VISUAL COMMUNICATION IN MEDICINE

<https://doi.org/10.1080/17453054.2023.2237087>

# Supplementary Material

## Supplementary Material A

Open-ended questionnaire results for the CG

| **Participant** | **Feedback** |
| --- | --- |
| CG1 | “*The resource didn't really address many of the questions in the quiz, but it was helpful data.”* |
| CG3 | *“the website didn't say anything about what these questions were asking, so it was confusing. it looked like a very technical site for research rather than something the general public would find informative.”* |
| CG4 | *“It is a mutation explorer website for research purposes only but if there could be a basic description of the function of each domain like the role of NTD, FCS etc. in the infection cycle then it would become more of a one stop place to understand everything being discussed. I felt that at one point I got slightly confused about the FCS but couldn't find any description on the website. The description need not be a detailed one, but just to brush up the memory of whoever is going through it.”* |
| CG5 | *“I am not sure my issue with the resource was usability per se - for my purposes, it would be a useful resource if I was looking for more information than, for example, a news site would give me about a specific mutation in a VOC they were talking about, but I'm not a virologist or medical expert, so most of the data doesn't mean a lot to me. You asked, in the question, about recognising various parts of the spike protein, but I didn't see anything in the website information explaining where these were located on it, in spite of the visualisations. I was able to infer a lot from the data and I think the website made raw data easier to navigate and visualise, so I think that worked very well, but "improvement" probably depends upon your audience. If you're targetting well-educated people with no experience in virology, it might help to have an additional page shortly explaining RBD/M, NTD, etc..., just so there's not information overload and the information is more accessible to the reader. If you're targetting experts, maybe this is all relatively apparent. The page DOES make the data more accessible, and I liked that the tables were sortable by field. I thought the visualisations were neat and probably would have been even cooler if I had more background knowledge.”* |
| CG6 | *“Since I suffer from color ametropia, coloring of the Pics was hard to detect (especially red from black, green from yellow...) I'd like to have had some menu positions named anywhere near the topics in those questions.”* |
| CG7 | *“i don't think this website is meant for the casual lay user. The only bits that made sense to me were the graphs showing rise and fall in the various mutations over time. My A level biology was of no use in understanding anything else and I didn't find any explanatory notes on the site for lay people but perhaps I didn't look hard enough.”* |
| CG8 | *“Liked the T cell epitope mutations data. Also, a suggestion, binding visualisation would help understand the concepts better.”* |

## Supplementary Material B

The experimental questionnaire


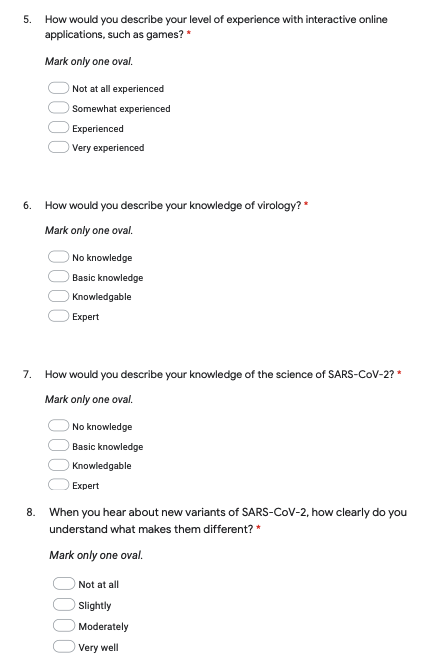

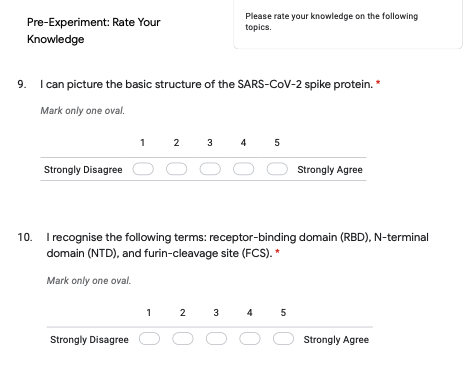

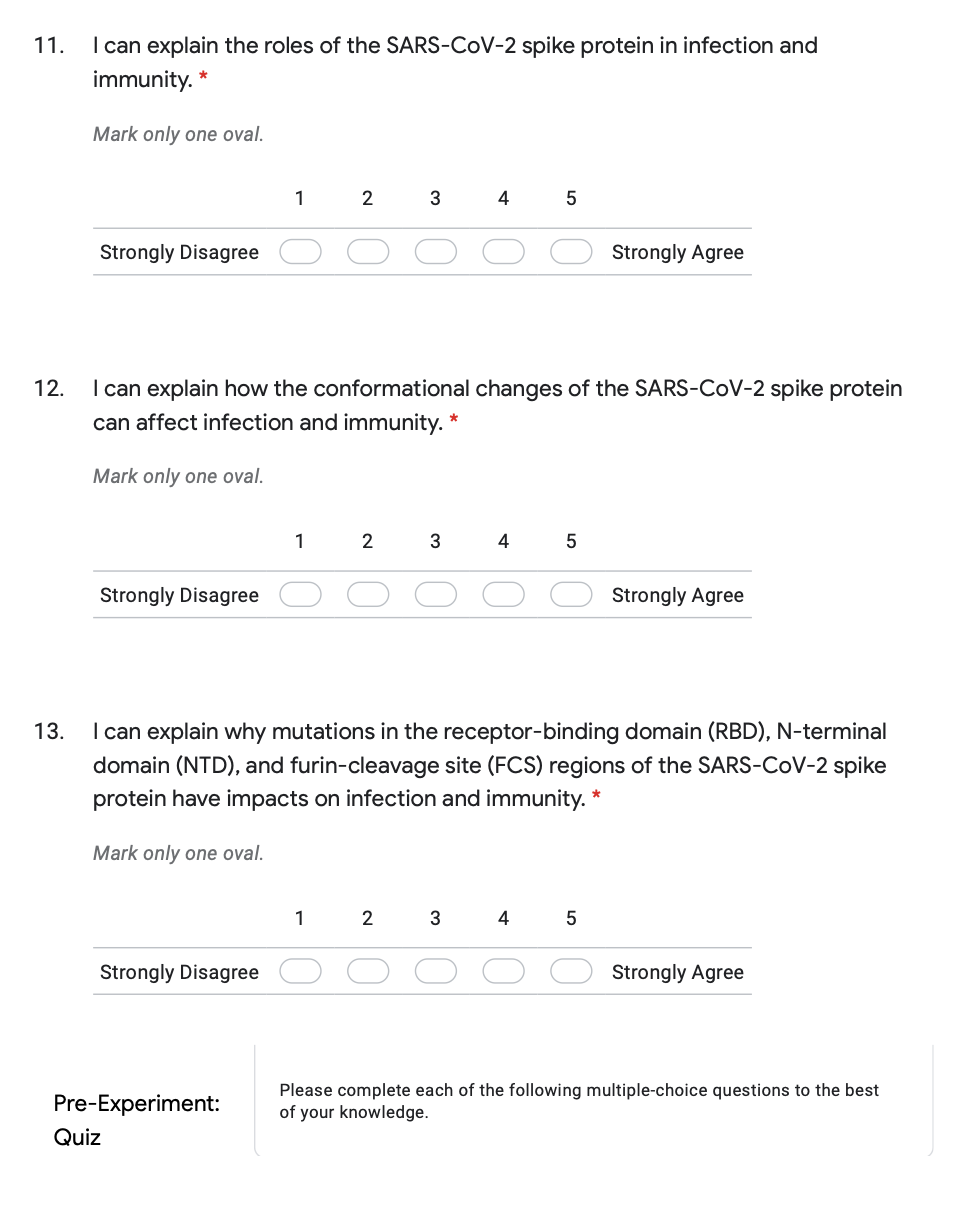

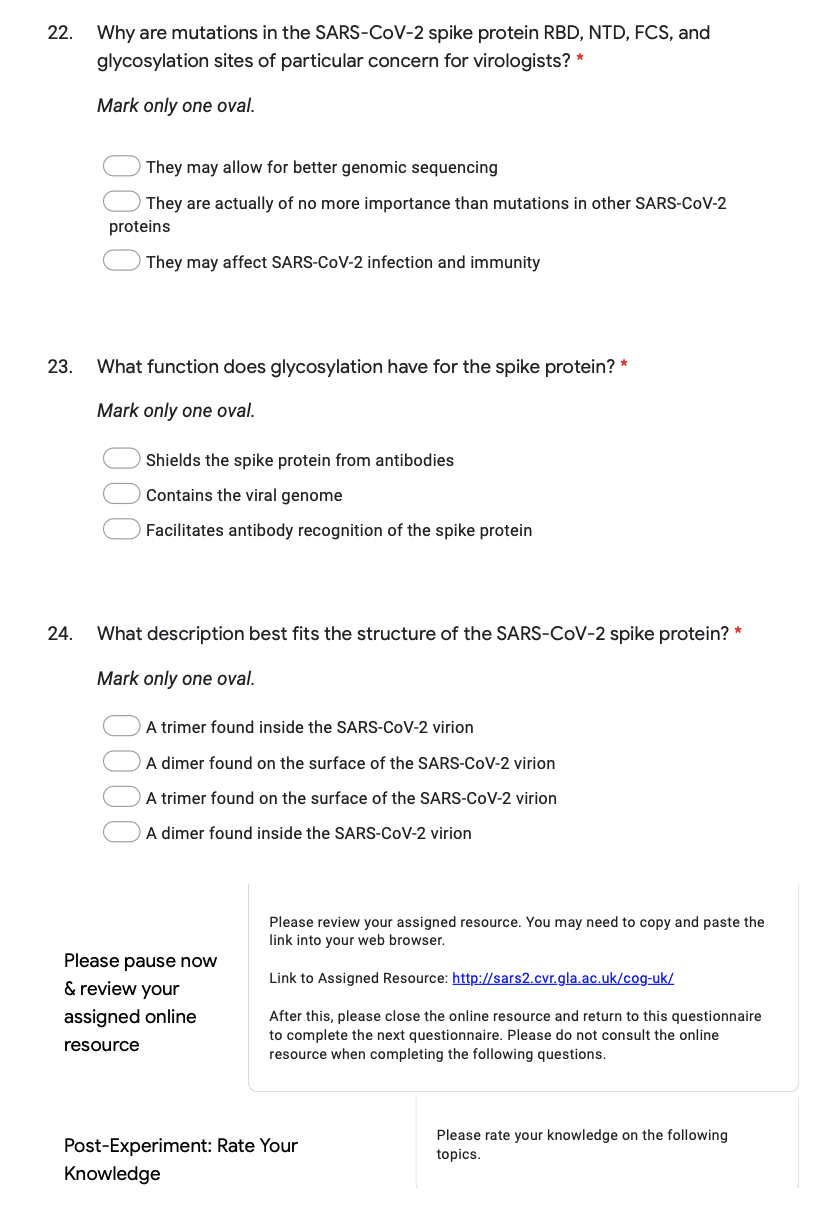

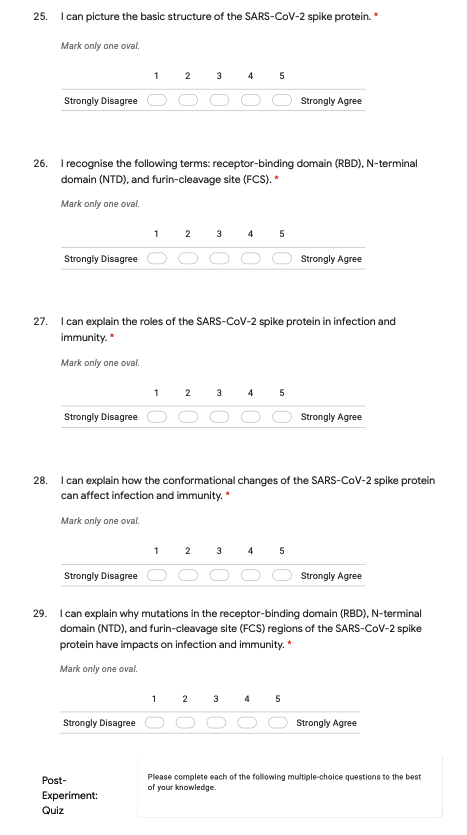

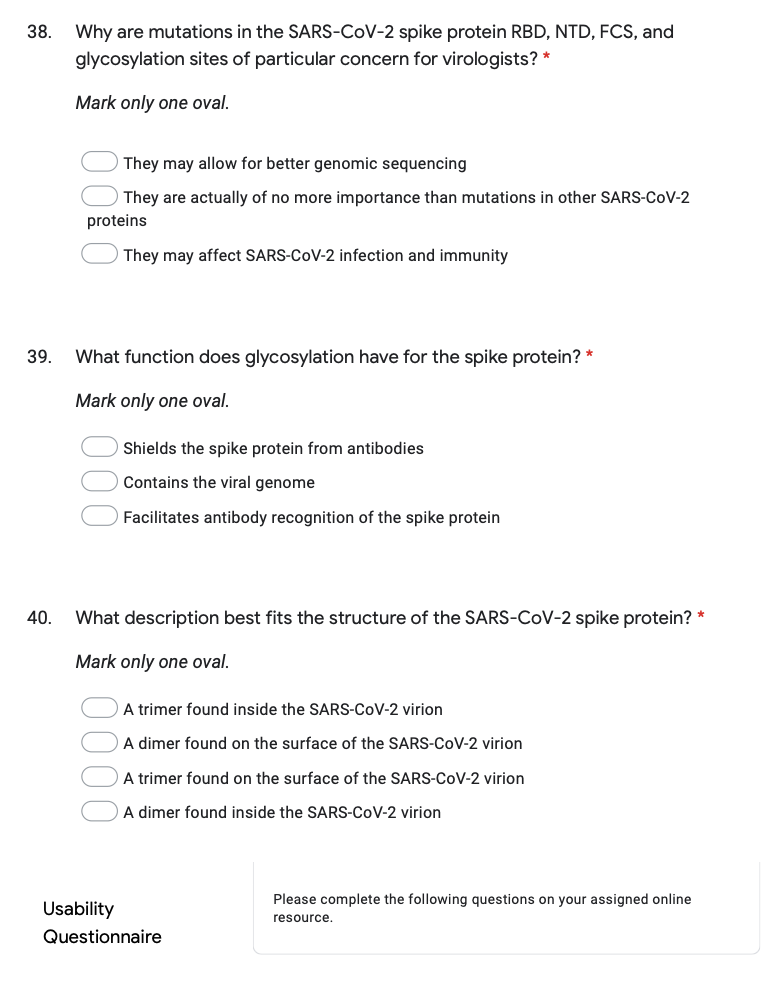

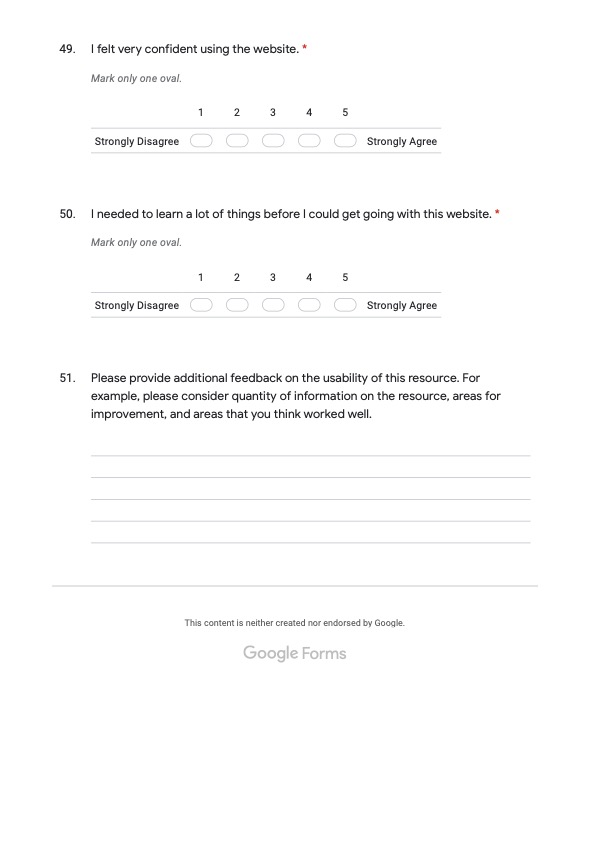


*Note: The Online resource review instructions and the Usability section were slightly altered for the Test Group due to the different nature of the resource.*


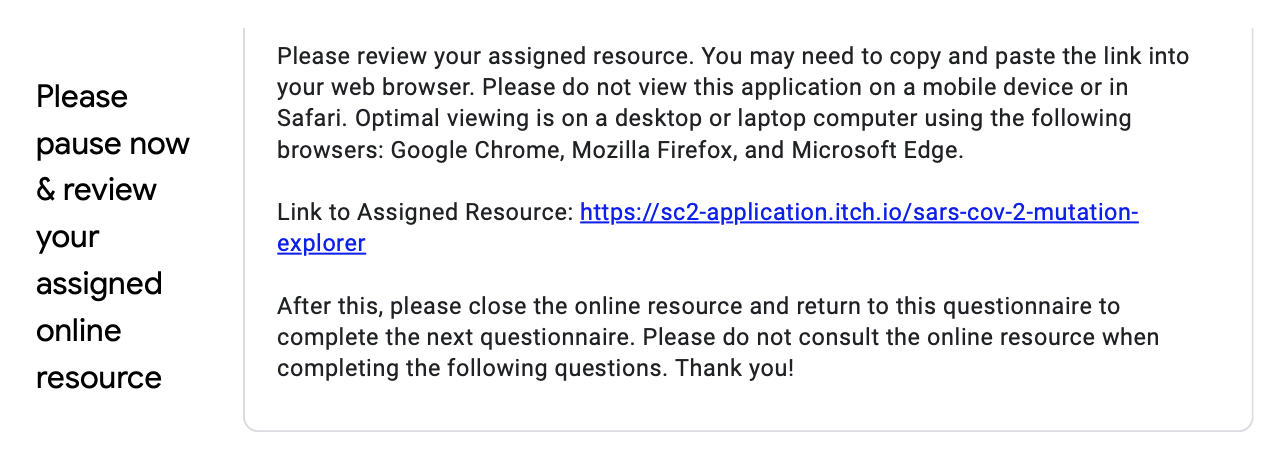

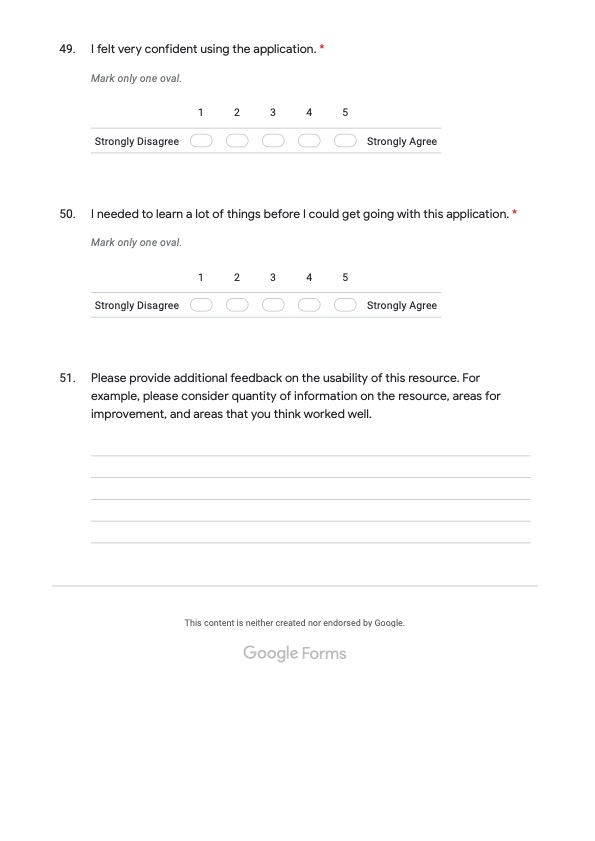


## Supplementary Material C

Open-ended questionnaire results for the TG

| **Participant** | **Feedback** |
| --- | --- |
| TG4 | *“Need to have more science background to understand all the terms used in this resource.”* |
| TG8 | *“When clicking in a part of the protein to read more about it, a window comes up but to return to the protein to check other parts I had to "Quit" the application and re-start it. It would be great if there is a way to just closed it and return to the protein.”* |
| TG9 | *“The resource is very nice. It is very easy to use it and to understand it.”* |
| TG10 | *“Easy to use, would be good to see open and closed conformations plus the effect of the cleavage sites to show the subunits together and then separate.”* |
| TG11 | *“Easy to implement changes: the video should be on the main page, not on specific pages, because it's a general video. There should be a progress bar on the video or at least a duration. Hyperlinks in the descriptions on words that are in the glossary could be of interest for users less familiar with biology. Scrolling down was very slow, but it could be my browser (Firefox).*  *Harder to do: the variants do not rotate, so it's harder to understand their specific mutations, compared to the general spike which is well represented. Obviously, the bets would be to see them added to the main structure, but that's probably a bit painful technically, especially for updates. The text describing their changes is in a very small window, makes for a lot of scrolling which is a bit tiresome.*  *On the whole, very nice project, those are just small suggestions to make it better. Well done!”* |
| TG12 | *“I crashed once while using, I was trying to go backwards. Sorry”* |
| TG14 | *“Voice record could be improved. Animation is very well. Information is clear. Easy to understand. Videos are understandable.”* |
